# Reported and Recorded Sleepiness in Obesity and Depression

**DOI:** 10.3389/fpsyt.2020.00200

**Published:** 2020-04-02

**Authors:** Juliane Minkwitz, Christian Sander, Hubertus Himmerich, Julia Thormann, Tobias Chittka, Ulrich Hegerl, Frank Schmidt, Monique Murray, Nihan Albayrak, Iain C. Campbell, Fabian Scheipl

**Affiliations:** ^1^Department of Translational Research in Psychiatry, Max Planck Institute of Psychiatry, München, Germany; ^2^IFB Adiposity Diseases, Leipzig University Medical Center, Leipzig, Germany; ^3^Department of Psychiatry and Psychotherapy, University Hospital Leipzig, Leipzig, Germany; ^4^Department of Psychological Medicine, King’s College London, London, United Kingdom; ^5^Department of Psychiatry, Psychosomatics and Psychotherapy, Goethe-Universität Frankfurt am Main, Frankfurt am Main, Frankfurt, Germany; ^6^Institute for Statistics, Ludwig-Maximilians-Universität München, München, Germany

**Keywords:** obesity, depression, EEG, sleepiness, VIGALL

## Abstract

**Background:**

Obesity and depression are both associated with changes in sleep/wake regulation, with potential implications for individualized treatment especially in comorbid individuals suffering from both. However, the associations between obesity, depression, and subjective, questionnaire-based and objective, EEG-based measurements of sleepiness used to assess disturbed sleep/wake regulation in clinical practice are not well known.

**Objectives:**

The study investigates associations between sleep/wake regulation measures based on self-reported subjective questionnaires and EEG-derived measurements of sleep/wake regulation patterns with depression and obesity and how/whether depression and/or obesity affect associations between such self-reported subjective questionnaires and EEG-derived measurements.

**Methods:**

Healthy controls (HC, N_HC_ = 66), normal-weighted depressed (DEP, N_DEP_ = 16), non-depressed obese (OB, N_OB_ = 68), and obese depressed patients (OBDEP, N_OBDEP_ = 43) were included from the OBDEP (Obesity and Depression, University Leipzig, Germany) study. All subjects completed standardized questionnaires related to daytime sleepiness (ESS), sleep quality and sleep duration once as well as questionnaires related to situational sleepiness (KSS, SSS, VAS) before and after a 20 min resting state EEG in eyes-closed condition. EEG-based measurements of objective sleepiness were extracted by the VIGALL algorithm. Associations of subjective sleepiness with objective sleepiness and moderating effects of obesity, depression, and additional confounders were investigated by correlation analyses and regression analyses.

**Results:**

Depressed and non-depressed subgroups differed significantly in most subjective sleepiness measures, while obese and non-obese subgroups only differed significantly in few. Objective sleepiness measures did not differ significantly between the subgroups. Moderating effects of obesity and/or depression on the associations between subjective and objective measures of sleepiness were rarely significant, but associations between subjective and objective measures of sleepiness in the depressed subgroup were systematically weaker when patients comorbidly suffered from obesity than when they did not.

**Conclusion:**

This study provides some evidence that both depression and obesity can affect the association between objective and subjective sleepiness. If confirmed, this insight may have implications for individualized diagnosis and treatment approaches in comorbid depression and obesity.

## Background

With over 1.9 billion adults affected worldwide (1), overweight and obesity cause significant negative health outcomes (2). Obesity is a well-known risk factor for somatic diseases such as diabetes, heart attacks, cancer, or sleep apnea (3). However, obesity also often co-occurs with mental illness, especially affective disorders like depression, and causes an enormous burden of disease (4) due to the deterioration in both the quality of life and the level of social functioning it entails.

Similar physiological processes are involved in the development and maintenance of both diseases (5). Nevertheless, associations between obesity and depression are very complex and not equally applicable to all people affected (6). This might be due to the fact that depression is a heterogenous disorder with multiple aetiological pathways interacting with genetic, neuroendocrinal, neurochemical, and neuroimmunlogical processes (7).

To further our understanding of how depression and obesity interact from a clinical perspective, the present paper is concerned with the link between depression and obesity focusing on sleep/wake regulation as a symptom complex that might connect both diseases, specifically, the occurrence and severity of disturbed sleep patterns. To do so, the following paragraphs provide precise definitions of brain arousal regulation, depression, atypical depression, and our operationalizations of these constructs before we describe our hypotheses in more detail.

According to the Diagnostic and Statistical Manual of Mental Disorders, Fifth Edition (DSM-5, 8), diagnosing a severe episode of depression requires five or more symptoms that can affect both the mental and physical well-being of the individual to persist for at least two weeks.

Symptoms include depressed mood, loss of interest, joylessness, feelings of worthlessness, and cognitive impairment, but also describe changes in activity levels, appetite, and sleep quality. Interestingly, the clinical manifestations of the heterogenous cohort of depressed patients differ particularly with regard to different characteristics of disturbances in sleep/wake regulation and appetite.

Therefore, atypical depression (AD) was included as a separate diagnosis in the fourth edition of Diagnostic and Statistical Manual for Mental Disorders in 1994 (DSM-IV, 9). AD defines a distinct symptom complex in depressed patients including mood reactivity, increased appetite and hypersomnia (10).

In fact, it is theorized that atypical depressed patients often suffer from significant weight gain or an increase in appetite with excessive sleep all through the day, whereas non-atypical depressed patients are characterized by significant weight loss or decrease in appetite and decreased sleep duration with early morning awakening. From a clinical perspective, varying symptoms with regard to sleep and appetite have a decisive influence on the choice of the individual treatment approach. Although a substantial body of literature already describes possible links between depression and body weight and/or sleep/wake regulation (e.g., 11–13), a deeper understanding of the connections between depression, obesity and sleep/wake regulation is likely to have important implications for individualized psychopharmacological and psychotherapeutic treatment of comorbid obesity and depression.

Sleepiness is the main consequence of insufficient sleep and dramatically influences the daytime brain arousal regulation. Brain arousal regulation, in turn, plays an important role in order to understand disturbed sleep/wake regulation in depression and describes a key process required to stabilize vigilance for higher cognitive performance. Vigilance as it is used here defines a tonic neurophysiologic arousal level rather than a function of attention. Besides, “arousal” has been defined as a relevant dimension investigating mental illness by the Research Domain Criteria project (14). Therefore, especially brain arousal regulation has recently become an important focus of interest in understanding the psychopathology of affective disorders.

The working group of Hegerl and colleagues defined the term EEG-vigilance in order to examine brain arousal regulation by means of electroencephalic activity (15). They found significantly more stable EEG-vigilance regulation in depressed patients compared to healthy controls and established the EEG-based “vigilance regulation model for affective disorders” (16).

The model hypothesizes that depressed patients (DEP) show less and later declines of brain arousal in resting state EEG recordings than healthy controls (HC). Thus, depressed patients are assumed to be characterized by a hyperstable EEG-vigilance regulation, whereas manic patients are assumed to have an unstable brain arousal regulation. Hegerl and colleagues (16, 17) characterize disturbed brain arousal regulation as a pathophysiological factor for depressive symptom complexes, such as sleep disturbances and daytime sleepiness. Furthermore, their model provides psychopathological explanations for, e.g., sensation avoidance and withdrawal in depression as a consequence of an autoregulatory attempt to handle hyperstable brain arousal.

In line with the predictions of the vigilance regulation model for affective disorders, several studies that apply electroencephalography-based approaches provide evidence for a hyperstable brain arousal regulation in depressed patients (18–20). Regarding disturbed sleep/wake regulation in depression, Hein and colleagues reported that approximately half of depressed patients suffer from excessive daytime sleepiness (EDS; 21) and that AD is a significant risk factor of EDS in major depression.

Moreover, previous findings of Bixler et al. (22) support the hypothesis that extreme daytime sleepiness (EDS) is more strongly associated with affective disorders and obesity than with sleep disordered breathing or sleep disruption per se. In a polysomnographic study, Fernandez-Mendoza et al. (23) showed that objective sleep disturbances predict incident daytime sleepiness in depressed individuals, whereas an increase in physiological sleep propensity predicts incident daytime sleepiness in those without depression.

Interestingly, results of a study by Plante et al. (24) indicate divergent associations between subjective and objective sleepiness in depression. Thus, self-reported sleep duration and EDS were positively associated with severity of depression, whereas objective sleepiness parameters were negatively associated with depressive symptoms.

Plante et al. (24) quantified objective sleepiness by the multiple sleep latency test (MSLT), which usually takes about 7 h in the course of a day and requires multiple devices. Although the MSLT is the gold standard for measuring daytime sleepiness, its applicability in a clinical setting is limited by its complex testing protocol. Moreover, the MSLT only assesses sleep onset, but does not provide information about the dynamics of brain arousal regulation before sleep onset. Therefore, measurements of resting state EEG activity constitute a preferable operationalization of objective sleepiness.

Resting state EEG activity is recorded typically for 15–20 min in eyes-closed condition and the dynamics of EEG-vigilance levels are recorded over time. To standardize this measurement, the working group of Hegerl and colleagues established an automatic tool for the classification of brain arousal regulation called VIGALL (Vigilance Algorithm Leipzig). VIGALL is an EEG- and electrooculography (EOG)-based algorithm which allows to objectively determine the level of EEG-vigilance and its dynamics within EEG recordings (25). It automatically classifies seven EEG-vigilance stages from wakefulness to varying degrees of drowsiness to sleep onset in a 1s-resolution (also see **Table 1**).

**Table 1:** Characteristics of EEG-vigilance stages according to VIGALL (Vigilance Algorithm Leipzig).

Brain arousal state	VIGALL		Score	EEG characteristics
High alertness	Stage 0		7	Low voltage EEG without α-activity, no horizontal SEM
Relaxed awake	Stage A	A1	6	Predominant occipital α-activity
		A2	5	High temporal and parietal α-activity
		A3	4	Predominant frontal α-activity
Drowsiness	Stage B	B1	3	Low voltage EEG without α-activity with horizontal SEM
		B2/3	2	Increase of δ- and θ-power
Sleep onset	Stage C		1	K-complexes and sleep spindles

## Aims and Hypotheses

To date, there exists no VIGALL-based study investigating brain arousal regulation in depressed patients with comorbid obesity, although the literature clearly indicates that both diseases affect sleep/wake regulation. Manifestations of sleep disturbances in depressed patients are complex and so far, it remains unclear how they are linked to obesity and subjective and objective sleepiness.

Moreover, based on the authors´ clinical expertise, obese depressed patients may subjectively suffer more often from severe daytime sleepiness and massive sleep disturbances than normal-weighted depressed patients. Nevertheless, it is currently unresolved whether such differences of subjective level of suffering can also be detected in objective EEG-based brain arousal regulation parameters or whether obese depressed patients assess subjective sleepiness differently and may overestimate the severity of disturbed sleep/wake regulation compared to normal-weighted depressed patients.

To shed some light on the psychopathological manifestation of disturbed sleep/wake regulation in depression and comorbid obesity, the aim of the present study is to investigate the relationship between subjective reported sleepiness and objective measured sleepiness in depressed and obese patients.

Therefore, a sample of healthy controls (HC), obese patients without depression (OB), depressed normal-weighted patients (DEP), and patients with depression and obesity (OBDEP) were examined via self-rating questionnaires and resting-state EEG recordings applying VIGALL.

We compared subjective parameters of sleepiness (subjective daytime sleepiness and subjective current states of sleepiness before and after EEG recordings) with objective EEG-based parameters of brain arousal regulation within and between these four subgroups.

Taken together, we hypothesize that obesity and depression affect the association between objective and subjective sleepiness.

## Methods

### Sample

As part of the OBDEP (Obesity and Depression) project volunteers were consecutively recruited between February 2011 and November 2012 from the outpatient clinic of the Integrated Research and Treatment Centre (IFB) for Adiposity Diseases Leipzig, from the Department of Psychiatry and Psychotherapy of the University Hospital Leipzig and via announcements (intranet, internet, local newspapers). One aim of the OBDEP project was to investigate the role of sleep and wakefulness regulation in the relationship between obesity and depression. The study was approved by University Leipzig Ethics Committee (#015–10–18012009).

The recruiting process for potential participants aged 18–70 years included a 20–40 min telephone interview to collect socio-demographic data plus a screening for somatic disorders and severe psychiatric conditions, using the German version of the checklist of the Structured Clinical Interview for DSM-IV (SKID; 26). Then, a full SCID-I was performed in cases of positive SCID screening.

The following inclusion criteria were defined: free from (a) psychiatric or neurological disorders other than depression, (b) history of head injury with loss of consciousness exceeding 1 h, (c) psychiatric medication, (d) acute or chronic infections, (e) current medication with a recognized major impact on the immune system, and (f) use of illegal drugs or abuse of alcohol within the past 6 months. After inclusion in the study, participants underwent a full physical examination by a study physician with weight and height measurements under standardized conditions.

The sample consists of N = 193 participants. We included N_HC_ = 66 healthy controls, N_OB_ = 68 obese patients without depression, N_DEP_ = 16 depressed normal-weighted patients, and N_OBDEP_ = 43 patients with comorbid depression and obesity.

### Subjective Sleepiness

We measured two subjective sleepiness parameters: daytime sleepiness [see *Day Time Sleepiness* Via *Epworth Sleepiness Scale (ESS)*] and situational sleepiness for a current state immediately before and after EEG recordings (see *Subjective Situational Sleepiness* Via *Self-Rating Questionnaires: KSS, SSS, VAS*).

#### Day Time Sleepiness via Epworth Sleepiness Scale (ESS)

The self-administered eight-item questionnaire “Epworth Sleepiness Scale (ESS)” was applied as it is proposed as a simple method for measuring subjective daytime sleepiness in adults (27) over the course of an entire day. Patients rate how likely they are to fall asleep in differing circumstances. Responders rate usual chances of dozing off or falling asleep while engaged in eight different everyday situations on 4-point scales with each item scored from 0 (not at all) to 3 (very likely). The ESS score can range from 0 to 24, whereas total scores of more than 10 points represent increasing levels of excessive daytime sleepiness (EDS). Internal consistency (Cronbach’s α: 0.73–0.86) of ESS is good, whereas external validity (0.11–0.43), and test-retest reliability (0.73–0.82) is described to be only weak to moderate (28).

#### Subjective Situational Sleepiness via Self-Rating Questionnaires: KSS, SSS, VAS

In addition to the ESS, participants were asked to rate their self-assessed current state of subjective sleepiness via the ordinal 1-item questionnaires Stanford Sleepiness Scale (SSS, 29) and Karolinska Sleepiness Scale (KSS; 30) before and after EEG recordings as both of these scales are sensitive to short-term fluctuations.

The KSS is a 10-point scale (1 = extremely alert to 10 = extremely sleepy, falls asleep all the time) and was found to be highly correlated to EEG and behavioral variables (30).

The SSS requires respondents to select one of seven (31) statements on a 7-point scale (1 = feeling active, vital, alert or wide awake to 7 = no longer fighting sleep, sleep onset soon, having dream-like thoughts) best representing their level of perceived current sleepiness. However, Akerstedt and colleagues (31, 32) criticize that several of the seven statements actually refer to fatigue or boredom rather than sleepiness.

As both SSS and KSS are applied in several recent studies for assessing self-reported sleepiness, we used both questionnaires for ordinal measurements of subjective sleepiness, although this might be redundant.

Historically, sleepiness was assessed using visual analogue scales (VAS, 33). Thus, we also used a VAS to evaluate the individual sleepiness of each participant on a metric scale from 0 (= not at all) to 100 (= very much).

SSS, KSS, and VAS were assessed twice. Score differences (ΔSSS, ΔKSS, ΔVAS) of measurements before (SSS_pre_/KSS_pre_/VAS_pre_) and after (SSS_post_/KSS_post_/VAS_post_) EEG recordings were calculated.

After the EEG recordings, participants were further asked to assess whether they had fallen asleep (variable ASLEEP) by choosing one of the four options: 1 = I definitely fell asleep, 2 = I may have fallen asleep, 3 = I probably did not fall asleep, 4 = I certainly did not fall asleep.

#### Covariates: Subjective Sleep Duration (SSD) and Subjective Sleep Quality (SSQ)

To control for confounding effects on subjective and objective sleepiness, we additionally assessed subjective sleep duration (SSD) of the last night before EEG recordings and subjective sleep quality (SSQ). SSQ is defined as the current degree of relaxation after last night sleep. SSQ was determined using the subscale “restedness” of a German sleep questionnaire SF-A (“Schlaffragebogen A”; 34), which consists of 8 items. Participants were asked to evaluate on a 5-point-scale (1 = not at all, 5 = very much) to what extent adjectives describing the sleep quality of the last night and well-being in the morning applied to them. SSQ was calculated by the quotient [sum score subscale “restedness”/8], where higher values correspond to higher degree of feeling relaxed after night sleep.

### Objective Sleepiness Parameter—EEG Vigilance Regulation

EEG analyses are a well-established approach for assessing levels of brain arousal and for monitoring sleep patterns. The EEG vigilance concept we refer to in our study relates unspecific activation patterns of the brain to a continuum from high alertness to sleep onset.

Early on, Loomis et al. (35) classified different states of cerebral activation on the basis of specific EEG patterns on a continuum ranging from a concentrated waking state to a deep sleep state. Bente (36) and Roth (37) further subdivided these EEG vigilance levels depending on the frequency and topographic distribution of the EEG waves (A1, A2, A3, B1, B2/3).

For an automatic classification of EEG vigilance, Hegerl and colleagues developed the computer-based algorithm VIGALL (Vigilance Algorithm Leipzig), which categorizes different EEG-vigilance stages on the basis of the frequency and topographical distribution of cerebral activity on a high temporal resolution. The algorithm was validated and further improved by simultaneous recordings of EEG and functional magnetic resonance imaging (fMRI) data (38) and by including EEG-power source estimates using sLORETA (standardized Low Resolution Brain Electromagnetic Tomography). VIGALL classifies each second of the EEG recording into one of seven EEG-vigilance stages ranging from high alertness (stage “0”), to relaxed wakefulness (stages “A1”, “A2”, “A3”), to drowsiness (stage “B1”, “B2/3”) up to sleep onset (stage “C”). **Table 1** defines EEG characteristics for each of these EEG-vigilance stages.

EEGs were recorded in eyes-closed condition for 20 min. All of the resting-state EEG recordings were taken between 08:00 and 18:00. They were recorded in a darkened and soundproofed room where participants sat in a comfortable chair in an upright position. The participants were instructed to keep their eyes closed, to relax and not to try to resist the urge to fall asleep.

EEG setup and recording: To record the EEG, 31 electrodes (sintered silver/silver chloride) with impedances below 10 kOhm were attached according to the international 10–20 system. The data were sampled at a rate of 1 kHz with a low-pass filter at 280 Hz, using the common average as a reference measure. An electrocardiogram (ECG) and an electrooculogram (EOG) were also recorded to monitor cardiac and ocular artifacts. For EOG recording, one electrode was placed on the forehead and a reference electrode on the cheek below the eye. The ECG electrodes were attached to the right and left wrist. The recordings were amplified with a 40-channel QuickAmp device and post-processed with BrainVision 2.0 software (BrainProducts, Gilching, Germany) installed on a Microsoft Windows XP-compatible computer system.

We then applied VIGALL to classify the EEG-vigilance stages at a 1s-resolution for each participant. Artefacted segments were excluded from further calculations based on established pre-processing protocols.

For more detailed information about EEG data recording and processing as well as operational methodology of VIGALL please refer to Huang et al. (39) and the VIGALL 2.1 manual (25).

Several objective sleep/wake regulation parameters were calculated on the basis of the VIGALL EEG-vigilance stages (18, 39).

- The proportion of time spent in each EEG-vigilance stage over 20 min.
- The mean vigilance values (MVV, range 1–7), i.e., averages of EEG-vigilance scores over 20 min, based on assigning a numeric value from 1 (lowest EEG-vigilance stage “C”) to 7 (highest EEG-vigilance stage “0”) to each EEG-vigilance stage.
- The criteria for the calculation of the arousal stability score (ASS, range 1–11) are described in **Table 2**.

**Table 2:** Criteria of the arousal stability score (ASS).

Score	Stability level criterion	Operational definition
11	less than 1/3 of all segments not classified as 0 or A/A1 stages	rigidity, only 0 and A1 stages
10		rigidity, only 0 and A stages
9	at least 1/3 of all segments classified as B stages	stage B emerged in minute 11–15
8		stage B emerged in minute 6–10
7		stage B emerged in 1–5
6	at least 1/3 of segments classified as B2/3 stages	stage B2/3 emerged in minute 11–15
5		stage B2/3 emerged in minute 6–10
4		stage B2/3 emerged in 1–5
3	occurrence of at least 1 C stage	stage C emerged in minute 11–15
2		stage C emerged in minute 6–10
1		stage C emerged in 1–5

### Obesity and Depression

Body mass index (BMI, [kg/m^2^]) was assessed according to WHO guidelines (40, 41). To assess symptoms and severity of depression, participants completed the revised Beck Depression Inventory, 2^nd^ edition (BDI-II; 42)

According to established cut-off values of BMI (40, 41) and BDI-II scores (43) participants were classified as: (1) HC = healthy controls (BMI < 30, BDI-II score < 14), (2) OB = obese non-depressed patients (BMI > 30, BDI-II score < 14), (3) DEP = normal-weighted, depressed patients (BMI < 30, BDI-II score > 13), and (4) OBDEP = obese depressed patients (BMI > 30, BDI-II score > 13).

### Statistics

Statistical analyses were performed using R version 3.5.3 (44) using packages cocor (45) and ordinal (46). Univariate between-group differences of location for metric and ordinal variables (see 2.1) were assessed using Kruskal-Wallis tests.

To assess the existence of a global association between subjective (SSS_pre_,KSS_pre_,VAS_pre_, SSS_post_,KSS_post_,VAS_post_, ΔSSS, ΔKSS, ΔVAS, ASLEEP) and objective (Proportions of VIGALL Stages 0, A, B, C, MVV, ASS) measures of sleepiness, canonical correlation analysis (CCA) between these two sets of variables was performed. The null hypothesis of no association was tested by means of the Pillai-Bartlett-Trace test (47), as implemented in R package CCP (48). The reported p-value for this test was computed based on a bootstrap approximation (9999 replications) of the null distribution to avoid unrealistic Gaussianity assumptions.

Bivariate associations of subjective measures of sleepiness or sleep quality and EEG vigilance-based measures (proportion of EEG-vigilance stages, MVV, ASS) were quantified via Spearman rank correlations and tested for significance as in Best and Roberts (49). Inter-group differences of group-specific estimated correlations were tested for significance based on Diedenhofen and Musch (45).

vRegression analyses of measures of subjective situational (VAS, KSS, ASS, sleep) or daytime sleepiness (ESS) were performed in order to assess between-group heterogeneity of their associations with objective EEG-vigilance based measures of situational sleepiness (MVV, ASS). These analyses corrected for the effects of age, gender, and sleep quality (SSQ) and included main effects as well as interaction effects of depression, obesity, and either MVV or ASS. The null hypothesis of homogeneity of association between subjective and objective measures of sleepiness across the groups defined by their (combined) depression and obesity status was assessed by testing for the presence of significant interaction effects between depression and/or obesity and the respective objective measure (MVV/ASS) on the respective subjective sleepiness parameters. For metric dependent variables (VAS, ESS), Gaussianity assumptions were validated using graphical regression model diagnostics and the significance of interaction effects was assessed by standard ANOVA F-tests. Proportional odds cumulative logit models (cf. 50) were used for ordinal dependent variables (KSS, SSS, ASLEEP) and the significance of interaction effects was assessed using Chi-Square likelihood ratio tests. Reported p-values were not corrected for multiple comparisons.

## Results

### Descriptive Statistics

Descriptive statistics for age, gender, SSD, SSQ, and subjective sleep parameters for all four subgroups are presented in **Tables 3A**–**C**.

**Table 3A:** Descriptive statistics (metric variables).

	HC (N=66)		OB (N=68)		DEP (N=16)		OBDEP (N=43)		
	Mean	SD	Mean	SD	Mean	SD	Mean	SD	p (Kruskal-Wallis)
Age	34	12	39	12	38	13	41	13	0.057
VAS (pre-EEG)	29	25	34	27	39	29	47	25	0.0039
VAS (post-EEG)	32	24	33	27	39	26	41	30	0.41
Δ VAS (post - pre)	3.3	26	-0.96	31	-0.31	37	-5.8	27	0.56
Δ KSS (post - pre)	0.52	1.4	-0.21	1.6	0	1.7	-0.4	1.7	0.0035
Δ SSS (post - pre)	0.21	1	-0.088	1.2	-0.19	1	-0.14	0.97	0.22
SSD (minutes)	432	69	427	81	462	74	436	75	0.44
SSQ	3.3	0.65	3.3	0.68	2.9	0.67	3	0.82	0.005
ESS	7.7	3.1	8.1	3.9	8.4	3.5	9.8	3.3	0.01

**Table 3B:** Descriptive statistics (categorical variables).

		HC (N=66)		OB (N=68)		DEP (N=16)		OBDEP (N=43)		
		N	%	N	%	N	%	N	%	p (χ^2^)
Gender	female	40	60.6	54	79.4	7	43.8	31	72.1	0.02
	male	26	39.4	14	20.6	9	56.2	12	27.9	
ASLEEP	definitively	9	13.6	5	7.4	1	6.2	9	20.9	0.11
	possibly	14	21.2	18	26.5	4	25	6	14	
	probably not	9	13.6	17	25	0	0	9	20.9	
	surely not	34	51.5	28	41.2	11	68.8	19	44.2	

**Table 3C:** Descriptive statistics (ordinal variables).

	HC (N=66)		OB (N=68)		DEP (N=16)		OBDEP (N=43)		
	Median	IQR	Median	IQR	Median	IQR	Median	IQR	p (Kruskal-Wallis)
KSS (pre-EEG)	3	3 – 4	3	3 – 5	4.5	3 – 6	5	3 – 6	0.0027
KSS (post-EEG)	4	3 – 5	3	2 – 4	4	3 – 5	4	3 – 6	0.12
SSS (pre-EEG)	2	2 – 2	2	1 – 3	3	2 – 4	3	2 – 4	9.4e-05
SSS (post-EEG)	2	2 – 3	2	2 – 3	3	2 – 3	2	2 – 4	0.0073

We included 132 female and 61 male participants in the study. The mean age of all subjects was M = 37.6 years (SD = 12.4 years). Subgroups differed in age (p =.057) and in terms of gender distribution (p =.016). Mean BDI-II scores were 4.4 (SD = 4.5) for HC, 5.4 (SD = 4.0) for OB, 24 (SD = 8.2) for DEP and 24 (SD = 8.8) for OBDEP. Mean BMI was 24 (SD = 2.9) for HC, 43 (SD = 7.5) for OB, 24 (SD = 3) for DEP, and 45 (SD = 8.9) for OBDEP.

Group differences were also found for SSQ (p =.005), although SSD did not differ between subgroups (p =.44).

#### Subjective Sleepiness.

Subgroups differed significantly in several subjective sleepiness parameters: self-reported daytime sleepiness (ESS, p =.010), self-reported sleepiness before (VAS_pre_, p =.004; KSS_pre_, p =.003; SSS_pre_, p < .001), and after EEG recordings (SSS_post_, p =.007) as well as in KSS score differences post and pre EEG (ΔKSS, p =.004), but did not differ in reported likelihood of having fallen asleep.

Moreover, we calculated correlations between subjective daytime sleepiness and subjective situational sleepiness. Overall, Spearman rank correlations were significantly positive between ESS – and SSS_pre_ (r =.26, p < .001), KSS_pre_ (r =.33, p < .001), VAS_pre_ (r =.25, p < .001), SSS_post_ (r =.19, p =.008), and KSS_post_ (r =.27, p < .001). Score differences after and before EEG recordings (ΔSSS/KSS/VAS) did not correlate significantly with ESS.

We further calculated correlations between subjective daytime/situational sleepiness and the covariate subjective sleep quality (SSQ). Overall, Spearman rank correlations were significantly negative between SSQ - and SSS_pre_ (r = −.44, p < .001), KSS_pre_ (r = −.45, p < .001), VAS_pre_ (r = −.42, p < .001), SSS_post_ (r = −.35, p < .001), KSS_post_ (r = −.34, p < .001), and VAS_post_ (r = −.29, p < .001). Score differences pre and post EEG recordings (ΔSSS/KSS/VAS) did not correlate significantly with SSQ. Furthermore, Spearman rank correlations between SSQ and ESS were significantly negative (r = −.28, p < .001).

#### Objective Sleepiness

Subgroups did not differ significantly in objective sleepiness parameters. Specifically, normal-weighted depressed and obese depressed patients did not differ significantly in objective sleepiness parameters according to results of the Wilcoxon rank sum test (MVV: p = 0.39; ASS: p = 0.7). Detailed information on distributions of EEG-vigilance stages, MVV and ASS are presented in **Table 3D**.

**Table 3D:** Distributions of EEG-vigilance stages, mean vigilance value (MVV), and arousal stability score (ASS).

	HC (N=66)		OB (N=68)		DEP (N=16)		OBDEP (N=43)		
	Mean	SD	Mean	SD	Mean	SD	Mean	SD	p (Kruskal-Wallis)
Proportion Stage 0	0.07	0.12	0.088	0.12	0.12	0.19	0.12	0.18	0.62
Proportion Stage A	0.47	0.3	0.48	0.33	0.54	0.31	0.47	0.32	0.85
Proportion Stage B	0.43	0.29	0.4	0.3	0.3	0.3	0.39	0.28	0.37
Proportion Stage C	0.012	0.044	0.012	0.048	0.012	0.033	0.0077	0.03	0.66
MVV	3.5	0.99	3.6	0.98	4	1.1	3.7	0.96	0.25
ASS	3.9	2.2	3.7	1.9	3.2	1.9	3.3	1.5	0.52

### Inference Statistics

#### Correlations Between Objective and Subjective Sleepiness Parameters

Canonical correlation analysis results indicate the existence of highly significant global association between subjective (SSS_pre_,KSS_pre_,VAS_pre_, SSS_post_,KSS_post_,VAS_post_, ΔSSS, ΔKSS, ΔVAS, ASLEEP) and objective (Proportions of VIGALL Stages 0, A, B, C, MVV, ASS) measures of sleepiness (Pillai-Bartlett-Trace test, p = 0.001). For ease of interpretation, we report the relevant bivariate objective-subjective correlations in the following.

##### Proportion of EEG Vigilance Stages

Correlations between the proportions of time spent in EEG-vigilance stages “0,” “A,” “B,” and “C,” respectively, and subjective sleepiness parameters were calculated.

Over all subjects, correlations revealed significant associations of the proportion of time spent in EEG-vigilance stages “A,” “B,” and “C” with the reported likelihood of having fallen asleep (“A”: r =.22, p =.002; “B”: r = −.31, p < .001; “C”: r = −.34, p < .001) and ESS (“A”: r = −.15, p =.04; “B”: r =.15, p =.035; “C”: r =.16, p =.026). In addition, proportion of time spent in stages “A” or “C” was associated (marginally) significantly with SSS_pre_ (“A”: −.14, p =.052; “C”:.18, p = 0.014). Furthermore, proportion of time spent in EEG-vigilance stage “A” was associated with pre-post changes in SSS (r =.15, p =.037) and proportion of time spent in EEG-vigilance stage “C” was associated significantly with both KSS_pre_ (r =.22, p =.002) and KSS_post_ (r =.2, p =.0062). None of the subjective sleepiness parameters were correlated significantly with proportion of time spent in EEG-vigilance stage “0.”

Correlations between EEG-vigilance stage proportions and subjective sleepiness parameters did not differ significantly between DEP and OBDEP. Significant correlation differences were found between DEP and HC for the correlations of time spent in EEG-vigilance stage “0” with pre-post changes in KSS (corr. difference:.55, p = 0.05), for the correlations of time spent in “A” with VAS_post_ (corr. difference: −.56, p = 0.05) and for the correlations of time spent in “B” with VAS_pre_ (corr. difference:.63, p =.02), VAS_post_ (corr. difference:.69, p =.01), and SSQ (corr. difference: −.61, p =.03). Significant correlation differences were found between OB and OBDEP for the correlations of time spent in stage B with KSS_pre_ (corr. difference: −.41, p =.03) and for the correlations of time spent in stage C with VAS_post_ (corr. difference:.46, p =.02) and pre-post changes in VAS (corr. difference:.48, p =.01).

**Table 4:** Correlation between EEG-based and subjective sleepiness, per subgroup.

EEG-Based	Subjective	HC	OB	DEP	OBDEP
MVV	SSS (pre-EEG)	−0.062	0.112	0.297	0.195
	KSS (pre-EEG)	0.040	0.058	0.230	0.435
	VAS (pre-EEG)	−0.103	0.029	0.498	0.160
	SSS (post-EEG)	−0.017	−0.079	0.450	−0.005
	KSS (post-EEG)	0.067	−0.009	0.276	0.213
	VAS (post-EEG)	−0.125	0.059	0.352	0.043
	Δ SSS (post - pre)	0.027	−0.206	0.135	−0.256
	Δ KSS (post - pre)	0.080	−0.042	0.115	−0.254
	Δ VAS (post - pre)	−0.029	−0.048	−0.173	−0.060
	ASLEEP	−0.301	−0.512	−0.517	−0.257
	ESS	0.034	0.127	0.322	0.148
	SSQ	0.099	−0.107	−0.438	−0.107
ASS	SSS (pre-EEG)	−0.062	0.112	0.297	0.195
	KSS (pre-EEG)	0.040	0.058	0.230	0.435
	VAS (pre-EEG)	−0.103	0.029	0.498	0.160
	SSS (post-EEG)	−0.017	−0.079	0.450	−0.005
	KSS (post-EEG)	0.067	−0.009	0.276	0.213
	VAS (post-EEG)	−0.125	0.059	0.352	0.043
	Δ SSS (post - pre)	0.027	−0.206	0.135	−0.256
	Δ KSS (post - pre)	0.080	−0.042	0.115	−0.254
	Δ VAS (post - pre)	−0.029	−0.048	−0.173	−0.060
	ASLEEP	−0.301	−0.512	−0.517	−0.257
	ESS	0.034	0.127	0.322	0.148
	SSQ	0.099	−0.107	−0.438	−0.107

##### MVV

Over all subjects, correlations revealed significant associations between MVV and the reported likelihood of having fallen asleep (r =.37, p < .001), MVV, and ESS (r = −.14, p =.05), and marginally significant associations between MVV and KSS_pre_ (r = −.13, p =.07).

Correlations between MVV and VAS_pre_ (correlation difference = −.66, p =.01) as well as between MVV and SSQ (correlation difference =.69, p =.01) differed significantly between HC and DEP. Correlations between objective and subjective sleepiness parameters did not differ significantly between DEP and OBDEP or between OB and OBDEP.

##### ASS

Over all subjects a significant correlation was found between ASS and the reported likelihood of having fallen asleep (r = −.37, p < .001).

Comparing HC and DEP, correlations between ASS and VAS_pre_ differed significantly (correlation difference =.60, p =.03), whereas correlations between ASS and SSQ (correlation difference = −.54, p =.06) differed marginally. Moreover, correlations between ASS and KSS_pre_ (r = −.38, p =.04) differed significantly between OB and OBDEP. No statistically significant correlation differences were found between DEP and OBDEP.

Correlations between objective and subjective sleepiness parameters for each subgroup are presented in **Table 4**.

#### ANOVAs

##### MVV/ASS and Subjective Situational Sleepiness (SSS; KSS, VAS)

###### Main Effects “age”

Regression analyses with either MVV or ASS as independent variables showed significant main effects of age on VAS_post_, ΔSSS, ΔKSS and ΔVAS (MVV: ΔVAS: F = 5.99, p =.015; ΔKSS: F = 12.80, p **< **.001; ΔSSS: F = 7.99, p **< **.001; VAS_post_: F = 16.60, p **< **.001; ASS: ΔVAS: F = 5.71, p =.018; ΔKSS: F = 13.30, p **< **.001; ΔSSS: F = 7.91, p =.006; VAS_post_: F = 16.10, p **< **.001). Moreover, regression analyses of MVV as independent variable revealed a significant main effect of age on KSS_post_(F = 4.99, p =.026), whereas regression analyses of ASS as dependent variable revealed a significant main effect of age on SSS_post_(F = 3.91, p =.048).

###### Main effect “SSQ”

Regression analyses with either MVV (ΔVAS: F = 4.50, p =.035; KSS_pre_: F = 5.92, p =.015; VAS_pre_: F = 39.80, p **< **.001; SSS_pre_: F = 40.90, p **< **.001; SSS_post_: F = 22.60, p **< **.001; VAS_post_: F = 13.80, p **< **.001; KSS_post_: F = 23.60, p **< **.001) or ASS (ΔVAS: F = 4.29, p =.040; KSS_pre_: F = 5.31, p =.022; VAS_pre_: F = 39.80, p **< **.001; SSS_pre_: F = 42.40, p **< **.001; SSS_post_: F = 24.10, p **< **.001; VAS_post_: F = 13.40, p **< **.001; KSS_post_: F = 23.50, p **< **.001) as independent variables revealed significant main effects of SSQ on almost all parameters of subjective situational sleepiness except ΔSSS and ΔKSS.

###### Main effect “gender”

Regression analyses with either MVV or ASS as independent variables revealed significant main effects of gender on ΔVAS (MVV: F = 4.92, p =.028, ASS: F = 4.69, p =.032) and KSS_pre_ (MVV: F = 5.92, p =.015, ASS: F = 6.19, p =.013) Regression analyses with MVV as independent variable further revealed a significant main effect of gender on ΔKSS (F = 4.92, p =.028).

###### Main effect “depression”

Regression analyses with either MVV (VAS_pre_: F = 6.49, p =.012; VAS_post_: F = 4.14, p =.043, KSS_pre_: F = 5.96, p =.015, SSS_pre_: F = 11.90, p **< **.001, SSS_post_: F =7.17, p =.007) or ASS (VAS_pre_: F = 5.31, p =.022; VAS_post_: F = 4.14, p =.043, KSS_pre_: F = 6.20, p =.013, SSS_pre_: F = 11.60, p **< **.001, SSS_post_: F = 7.74, p =.005) as independent variables revealed a significant main effect of depression on almost all parameters of subjective situational sleepiness pre and post EEG recordings, except KSS_post_, but not on score differences of subjective sleepiness (ΔSSS, ΔKSS, ΔVAS).

###### Main effect “obesity”

Main effects of obesity on VAS_pre_ were found to be significant (MVV: F = 3.76, p =.054; ASS: F = 3.85, p =.051). Regression analyses of MVV as dependent variable revealed a significant main effect of obesity on ΔKSS (F = 4.09, p =.045). Furthermore, a significant main effect of obesity (F = 3.73, p =.053) on ASLEEP was found.

###### Associations between subjective and objective sleepiness parameters

Moreover, regression analyses revealed a significant association between MVV and ASLEEP (F = 23.40, p **< **.001) as well as between ASS and ASLEEP (F = 27.90, p **< **.001).

###### Interaction Effects

Regression analyses revealed a marginally significant interaction effect of depression on the association of MVV with VAS_pre_ (F = 3.73, p =.055).

Moreover, results showed a significant interaction effect of obesity on the association between of ASS with VAS_pre_ (F = 2.79, p =.042).

##### MVV/ASS and Subjective Daytime Sleepiness

###### Main Effects “SSQ”

Main effects of **SSQ** on ESS (F= 15.30, p **< **.001) were found to be significant.

###### Main effect “depression”

Regression analyses with MVV as independent variable revealed a significant main effect of depression on ESS (F = 4.84, p =.029).

###### Associations between subjective and objective sleepiness parameters

Moreover, regression analyses revealed a marginally significant association between MVV and ESS (F = 3.68, p =.057).

###### Interaction Effects

Regression analyses revealed no interaction effects on the association between objective sleepiness and subjective daytime sleepiness.

## Discussion

In the present study, we investigated the relationship between obesity, depression, subjective reported sleepiness, and objective measured sleepiness.

To investigate this, we examined reported daytime and situational sleepiness as well as objectively measured EEG-based sleepiness parameters in normal-weighted depressed, obese depressed, obese non-depressed patients, and healthy controls.

Generally speaking, subjective sleepiness parameters differed significantly between the four subgroups, whereas objective sleepiness parameters did not. Similar phenomena have previously been reported by Denton et al. (51), who reported weak correlation only between subjective but not objective sleepiness and affective disorders and also by Plante et al. (24, 52) who reported divergent associations between subjective and objective measures of hypersomnolence and depression. In addition, the fact that “[…] only objective but not subjective sleepiness was significantly associated with the reported likelihood of having fallen asleep during EEG recordings [… could suggest that some] patients may have rated depression related tiredness or apathy as sleepiness,” as one reviewer put it.

Descriptive analyses showed that both subjective daytime sleepiness and subjective sleep quality were associated with reported situational sleepiness. However, directions and magnitudes of changes in reported sleepiness before and after EEG recordings did not vary systematically with subjective daytime sleepiness or with subjective sleep quality. Counterintuitively, 20 min of resting in eyes-closed condition seem to change reported situational sleepiness pre and post EEG recordings in the same way regardless of subjective daytime sleepiness or reported sleep quality.

Furthermore, ANOVA results provide evidence that both depression and obesity are associated with subjective sleepiness.

While depression significantly affects both subjective daytime sleepiness, i.e., ESS, and almost all parameters of reported situational sleepiness pre and post EEG recordings, obesity does not seem to be associated with subjective daytime sleepiness and only with reported situational sleepiness before EEG recordings when evaluated using the metric visual analogue scale.

These findings partly contradict the results of previous studies exploring the role of obesity in excessive daytime sleepiness. Thus, a study by Slater and colleagues (53) identified obesity as an independent predictor for subjective daytime sleepiness. Similarly, Maugeri and colleagues (54) investigated a non-depressed cohort and found short sleep duration and EDS to be associated with greater odds of overweight and obesity, independent of diet and physical activity.

Moreover, ANOVA results show that both depression and obesity affect the association between objective measured sleepiness and subjective reported sleepiness before EEG recordings when assessed by the metric visual analogue scale, but not when assessed by KSS or by SSS.

To summarize: In the present study, reported situational sleepiness before EEG recordings was associated with obesity only when the former was assessed by the metric VAS. Similarly, depression and obesity were found to affect the association between objective measured sleepiness and subjected reported sleepiness before EEG recordings only when the latter was assessed by the metric VAS. We now discuss two possible explanations for this surprising result, which may also be related to methodological shortcomings of our study.

As participants were asked to evaluate their situational sleepiness simultaneously on a metric scale (VAS) displayed as a horizontal line with a range from 0 to 100 on which the participants mark how tired they are, and on two ordinal scale (KSS and SSS), this triple query may distort the recorded responses. KSS and SSS are very similar questionnaires requiring responders to select one of 10 (KSS) or 7 (SSS) statements about their situational sleepiness. Responding to KSS and SSS shortly after one another may cause cognitive dissonance avoidance behavior among the participants, in the sense that they may compare their answers to KSS and SSS in order to choose maximally consistent statements.

Therefore, KSS and SSS scores could be biased by a kind of social desirability bias, as participants may try to give reliable and valid answers. In contrast, VAS scoring might be more intuitive and easier to choose compared to KSS and SSS, where participants carefully weigh up, and potentially attempt to compare, the 7 or 10 statements that differ only slightly from each other.

Additionally, and more generally, another methodological drawback of our study is due to the fact that different concepts of sleepiness, fatigue and exertion coexist and might be cross-associated (55). Therefore, it is questionable whether both SSS and KSS are suitable for validly measuring situational sleepiness. To our knowledge, no studies exist that compare the reliability and validity of SSS and KSS to assess situational sleepiness. Taken together, the VAS is likely to provide more reliable and valid measurements to evaluate situational sleepiness.

Further results show that objective measured sleepiness was significantly associated with the reported likelihood of having fallen asleep during EEG recordings and was almost significantly associated with subjective daytime sleepiness. These results are in line with recent findings of a study by Jawinski et al. (56), which investigated the association between recorded and reported sleepiness in a population-based cohort study including 10,000 randomly selected inhabitants of Leipzig, Germany. They found moderate correlations between objective sleepiness parameters and the reported likelihood of having fallen asleep.

Although analyses revealed no significant correlation differences of objective and subjective sleepiness parameters between normal-weighted depressed (DEP) and obese depressed patients (OBDEP), we can report, nevertheless, that correlations in the OBDEP subgroup were systematically weaker than in the DEP subgroup (see **Table 4**).

Interestingly, while normal-weighted depressed patients (DEP) and healthy controls (HC) differ significantly with regard to the strengths and directions of correlations between some subjective sleepiness parameters and the objective sleepiness parameter MVV, our results show that strengths and directions of correlations between objective and subjective sleepiness parameters are very similar in the obese non-depressed (OB) and obese depressed subgroup (OBDEP). Thus, in the normal-weighted subgroup, this study provides some evidence that depression affects the association between subjective and objective sleepiness, but not in the obese subgroup.

This pattern is much less clear for correlations between the objective sleepiness parameter ASS and subjective sleepiness parameters. Indeed, this might be due to the fact that ASS is designed to measure dynamic aspects of brain arousal regulation and not just mean situational sleepiness over a 20-min time span. Therefore, the functional course of changing EEG-vigilance over 20 min captured by ASS may conceptually define a different construct than situational sleepiness itself at a certain measure time before and after EEG recordings, as these constructs are based on different temporary conditions and may describe not just current states, but also traits of long term individual characteristics.

Thus, MVV is likely to be a valid measurement for objective situational sleepiness, whereas ASS may be better suited to describe brain arousal regulation according to the vigilance regulation model for affective disorders. Accordingly, we conclude that brain arousal regulation and consequently objective sleepiness might be more complex in depressed patients, especially with comorbid obesity, and should therefore be examined again more closely by means of more precise statistical methods. In line with such potentially complex associations of sleep/wake regulation with depression, a study by Geoffrey and colleagues (57) analyzing a nationally representative survey of the US adult population showed that insomnia sleep patterns and hypersomnia often co-occur in depressed patients. As our results show, controlling for additional covariates such as obesity may help to provide further insight into such phenomena in the depressed cohort. In a similar vein, further studies should also assess more detailed clinical characteristics of depressive symptoms to distinguish between depression and depression with atypical features in order to improve our understanding of these associations.

However, the small sample size, especially of the normal-weighted depressed subgroup is a drawback, which complicates the interpretation of the findings.

Furthermore, the present study and previous studies on brain arousal regulation using VIGALL only analyzed minute-wise pseudo-means of the ordinal high-frequency EEG-vigilance stages and fairly ad-hoc summaries of their evolution over time, which may obscure the complexity of brain arousal regulation dynamics. Taken together, for more precise analyses of both objective sleepiness and brain arousal regulation dynamics in depressed patients and their association with or confounding by comorbidities like obesity, future studies should take functional dynamics of the EEG-vigilance stages over the whole recording period in the original 1s-resolution into account in order to avoid the loss of information incurred by simply averaging EEG-segments over time (MVV) or merely analyzing first-occurrence times of lower EEG-vigilance stages and coarse categorizations of vigilance stage distributions over time (ASS).

## Conclusion

This study provides some evidence that both depression and obesity may affect the association between objective and subjective sleepiness.

If confirmed, this insight might have future implications for individualized diagnosis and treatment approaches in comorbid depression and obesity. For example, depressive syndromes related to sleep/wake regulation may affect treatment decisions with regard to different substance classes of antidepressants that take into account their sleep-inducing effects and/or their effects on patients´ activity levels.

This clinical decision should also consider that the subjective assessment of sleep/wake regulation may be affected by obesity, as the present study indicates. To achieve optimal treatment choices, then, it might be beneficial to rely on more objective measures based on the resting state EEG recordings, which are broadly available in a clinical setting and increasingly part of the standard set of diagnostics for depressed patients. Such VIGALL-based diagnostics are a more economic and time-efficient choice that is easier to perform in clinical settings than the MSLT.

## Data Availability Statement

The datasets generated for this study are available to be shown on request to the corresponding author.

## Ethics Statement

The studies involving human participants were reviewed and approved by the University Leipzig Ethics Committee (#015–10–18012009). The patients/participants provided their written informed consent to participate in this study.

## Author Contributions

HH, CS, UH, and JM contributed to the conception and design of the study. JM, JT, TC, HH, and FrS collected the data. MM, NA, IC, JT, TC, CS, FaS, and JM organized the data base. CS, JT, TC, FrS, UH, and JM carried out the EEG-analyses. FaS and JM performed statistical analyses. FaS summed up the results and created the tables. JM wrote the first draft of the manuscript. IC, MM, NA, and FaS improved the language of the manuscript. All authors contributed to manuscript revision, and read and approved the submitted version.

## Funding

This study was supported by the Integrated Research and Treatment Centre for Adiposity Diseases (IFB), University of Leipzig, the Federal Ministry of Education and Research (BMBF), FKZ: 01EO1001. Publication costs were covered by the Max-Planck-Society. The funding sources had no role in the design and conduct of the study; collection, management, analysis, and interpretation of the data; and preparation, review, or approval of the manuscript. Hubertus Himmerich has received salary support from the National Institute for Health Research (NIHR) Biomedical Research Centre (BRC) at South London and Maudsley NHS Foundation Trust (SLaM) and King’s College London.

## Conflict of Interest

The authors declare that the research was conducted in the absence of any commercial or financial relationships that could be construed as a potential conflict of interest.
